# 3-Cyclo­hexyl­sulfonyl-2-methyl-5-propyl-1-benzofuran

**DOI:** 10.1107/S1600536811031023

**Published:** 2011-08-06

**Authors:** Pil Ja Seo, Hong Dae Choi, Byeng Wha Son, Uk Lee

**Affiliations:** a Department of Chemistry, Dongeui University, San 24 Kaya-dong Busanjin-gu, Busan 614-714, Republic of Korea; b Department of Chemistry, Pukyong National University, 599-1 Daeyeon 3-dong, Nam-gu, Busan 608-737, Republic of Korea

## Abstract

In the title compound, C_18_H_24_O_3_S, the cyclo­hexyl ring adopts a chair conformation. In the crystal, mol­ecules are linked through weak inter­molecular C—H⋯O hydrogen bonds and C—H⋯π inter­actions. In the propyl group, one C atom is disordered over two sites with site-occupancy factors of 0.546 (8) and 0.454 (8).

## Related literature

For the pharmacological activity of benzofuran compounds, see: Aslam *et al.* (2009 ▶); Galal *et al.* (2009 ▶); Khan *et al.* (2005 ▶). For natural products with benzofuran rings, see: Akgul & Anil (2003 ▶); Soekamto *et al.* (2003 ▶). For structural studies of related 5-alkyl-3-cyclo­hexyl­sulfonyl-2-methyl-1-benzofuran derivatives, see: Choi *et al.* (2011 ▶); Seo *et al.* (2011 ▶).
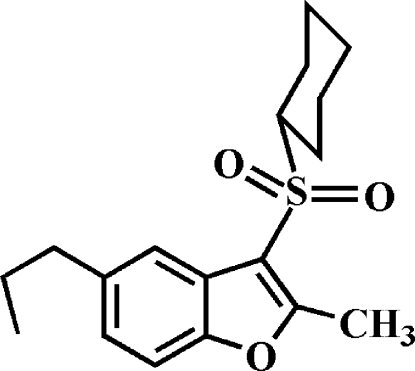


## Experimental

### 

#### Crystal data

C_18_H_24_O_3_S
*M* *_r_* = 320.43
Monoclinic, 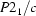

*a* = 5.735 (2) Å
*b* = 23.713 (9) Å
*c* = 12.618 (5) Å
β = 100.389 (10)°
*V* = 1687.7 (11) Å^3^
*Z* = 4
Mo *K*α radiation
μ = 0.20 mm^−1^
*T* = 173 K
0.36 × 0.11 × 0.09 mm

#### Data collection

Bruker SMART APEXII CCD diffractometer
Absorption correction: multi-scan (*SADABS*; Bruker, 2009 ▶) *T*_min_ = 0.931, *T*_max_ = 0.982
14906 measured reflections
3644 independent reflections
2370 reflections with *I* > 2σ(*I*)
*R*_int_ = 0.081

#### Refinement

*R*[*F* ^2^ > 2σ(*F* ^2^)] = 0.061
*wR*(*F* ^2^) = 0.158
*S* = 1.06
3644 reflections
204 parameters
18 restraints
H-atom parameters constrained
Δρ_max_ = 0.47 e Å^−3^
Δρ_min_ = −0.39 e Å^−3^

### 

Data collection: *APEX2* (Bruker, 2009 ▶); cell refinement: *SAINT* (Bruker, 2009 ▶); data reduction: *SAINT*; program(s) used to solve structure: *SHELXS97* (Sheldrick, 2008 ▶); program(s) used to refine structure: *SHELXL97* (Sheldrick, 2008 ▶); molecular graphics: *ORTEP-3* (Farrugia, 1997 ▶) and *DIAMOND* (Brandenburg, 1998 ▶); software used to prepare material for publication: *SHELXL97*.

## Supplementary Material

Crystal structure: contains datablock(s) global, I. DOI: 10.1107/S1600536811031023/xu5281sup1.cif

Structure factors: contains datablock(s) I. DOI: 10.1107/S1600536811031023/xu5281Isup2.hkl

Supplementary material file. DOI: 10.1107/S1600536811031023/xu5281Isup3.cml

Additional supplementary materials:  crystallographic information; 3D view; checkCIF report

## Figures and Tables

**Table 1:** Hydrogen-bond geometry (Å, °) *Cg* is the centroid of the C2–C7 benzene ring.

*D*—H⋯*A*	*D*—H	H⋯*A*	*D*⋯*A*	*D*—H⋯*A*
C13—H13⋯O3^i^	1.00	2.34	3.314 (3)	164
C18—H18*A*⋯O2^ii^	0.99	2.51	3.345 (4)	142
C12—H12*C*⋯*Cg*^iii^	0.98	2.84	3.659 (3)	141

Symmetry codes: (i) 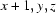; (ii) 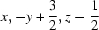; (iii) 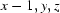.

**Fig. 1.**
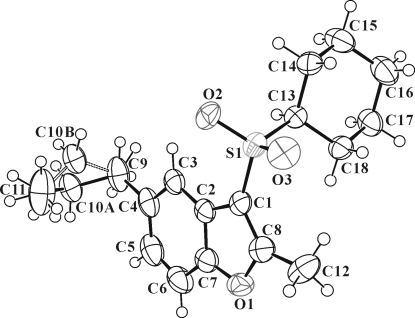
The molecular structure of the title compound with the atom numbering scheme. Displacement ellipsoids are drawn at the 50% probability level. H atoms are presented as small spheres of arbitrary radius. The C10 atom of the propyl group is disordered over two positions with site occupancy factors, from refinement of 0.546 (8) (part A) and 0.454 (8) (part B).

**Fig. 2.**
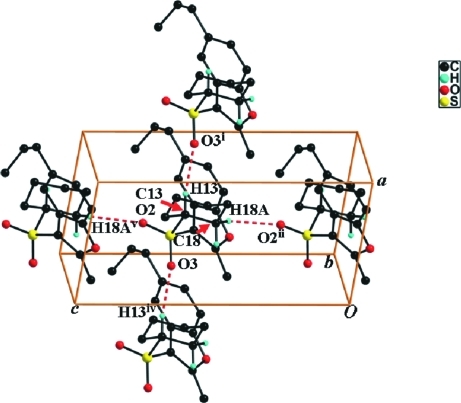
A view of the C—H···O and C—H···π interactions (dotted lines) in the crystal structure of the title compound. [Symmetry codes: (i) x + 1, y, z; (ii) x, - y + 3/2, z - 1/2; (iv) x - 1, y, z; (v) x, - y + 3/2, z + 1/2.]

**Fig. 3.**
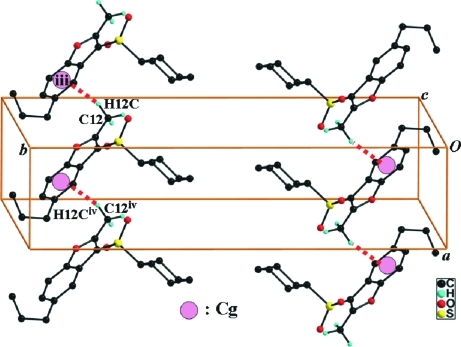
A view of the C—H···π interactions (dotted lines) in the crystal structure of the title compound. [Symmetry codes: (iii) x - 1, y, z; (vi) - x + 2, - y + 1, - z + 1.]

## supplementary crystallographic information

### Comment

Recently, many compounds involving a benzofuran ring have received much attention due to their valuable pharmacological properties such as antibacterial and antifungal, antitumor and antiviral, and antimicrobial activities (Aslam *et al.*, 2009, Galal *et al.*, 2009, Khan *et al.*, 2005). These benzofuran derivatives occur in a wide range of natural products (Akgul & Anil, 2003; Soekamto *et al.*, 2003). As a part of our continuing studies of the substituent effect on the solid state structures of 5-alkyl-3-cyclohexylsulfonyl-2-methyl-1-benzofuran analogues (Choi *et al.*, 2011; Seo *et al.*, 2011), we report herein the crystal structure of the title compound.

In the title molecule (Fig. 1), the benzofuran unit is essentially planar, with a mean deviation of 0.008 (2) Å from the least-squares plane defined by the nine constituent atoms. The cyclohexyl ring is in the chair form. In the propyl group, C10 atom is disordered over two positions with site occupancy factors, from refinement of 0.546 (8) (part A) and 0.454 (8) (part B). The molecular packing (Fig. 2) is stabilized by weak intermolecular C—H···O hydrogen bonds; the first one between a cyclohexyl H atom and the O atom of the sulfonyl group (Table 1; C13—H13···O3^i^), and the second one between a cyclohexyl H atom and the O atom of the sulfonyl group (Table 1; C18—H18A···O2^ii^). The crystal packing (Fig. 3) is further stabilized by intermolecular C—H···π interactions between a methyl H atom and the benzene ring (Table 1; C12—H12C···Cg^iii^, Cg is the centroid of the C2–C7 benzene ring).

### Experimental

77% 3-Chloroperoxybenzoic acid (560 mg, 2.5 mmol) was added in small portions to a stirred solution of 3-cyclohexylsulfanyl-2-methyl-5-propyl-1-benzofuran (346 mg, 1.2 mmol) in dichloromethane (40 mL) at 273 K. After being stirred at room temperature for 8 h, the mixture was washed with saturated sodium bicarbonate solution, and the organic layer was separated, dried over magnesium sulfate, filtered and concentrated at reduced pressure. The residue was purified by column chromatography (hexane–ethyl acetate, 4:1 v/v) to afford the title compound as a colorless solid [yield 70%, m.p. 393–395 K; *R*_f_ = 0.60 (hexane–ethyl acetate, 4:1 v/v)]. Single crystals suitable for X-ray diffraction were prepared by slow evaporation of a solution of the title compound in benzene at room temperature.

### Refinement

All H atoms were positioned geometrically and refined using a riding model, with C—H = 0.95 Å for aryl, 1.00 Å for methine, 0.99 Å  for methylene and 0.98 Å for methyl H atoms, respectively. U_iso_(H) = 1.2*U*_eq_(C) for aryl, methine, methylene and 1.5*U*_eq_(C) for methyl H atoms. The C10 atom of the propyl group is disordered over two positions with site occupancy factors, from refinement of 0.546 (8) (part A) and 0.454 (8) (part B). The C—C distance sets were restrained to 0.001 Å using command SADI, and displacement ellipsoids of C10A and C10B set were restrained to 0.01 using command ISOR, EADP and DELU.

### Crystal data

**Table:**

C_18_H_24_O_3_S	*F*(000) = 688
*M**_r_* = 320.43	*D*_x_ = 1.261 Mg m^−^^3^
Monoclinic, *P*2_1_/*c*	Mo *K*α radiation, λ = 0.71073 Å
Hall symbol: -P 2ybc	Cell parameters from 2565 reflections
*a* = 5.735 (2) Å	θ = 3.1–26.3°
*b* = 23.713 (9) Å	µ = 0.20 mm^−^^1^
*c* = 12.618 (5) Å	*T* = 173 K
β = 100.389 (10)°	Block, colourless
*V* = 1687.7 (11) Å^3^	0.36 × 0.11 × 0.09 mm
*Z* = 4	

### Data collection

**Table:**

Bruker SMART APEXII CCD diffractometer	3644 independent reflections
Radiation source: rotating anode	2370 reflections with *I* > 2σ(*I*)
graphite multilayer	*R*_int_ = 0.081
Detector resolution: 10.0 pixels mm^-1^	θ_max_ = 27.0°, θ_min_ = 1.7°
φ and ω scans	*h* = −7→7
Absorption correction: multi-scan (*SADABS*; Bruker, 2009)	*k* = −27→29
*T*_min_ = 0.931, *T*_max_ = 0.982	*l* = −15→16
14906 measured reflections	

### Refinement

**Table:**

Refinement on *F*^2^	Primary atom site location: structure-invariant direct methods
Least-squares matrix: full	Secondary atom site location: difference Fourier map
*R*[*F*^2^ > 2σ(*F*^2^)] = 0.061	Hydrogen site location: difference Fourier map
*wR*(*F*^2^) = 0.158	H-atom parameters constrained
*S* = 1.06	*w* = 1/[σ^2^(*F*_o_^2^) + (0.0602*P*)^2^ + 0.1356*P*] where *P* = (*F*_o_^2^ + 2*F*_c_^2^)/3
3644 reflections	(Δ/σ)_max_ < 0.001
204 parameters	Δρ_max_ = 0.47 e Å^−^^3^
18 restraints	Δρ_min_ = −0.39 e Å^−^^3^

### Special details

**Table:**

Geometry. All esds (except the esd in the dihedral angle between two l.s. planes) are estimated using the full covariance matrix. The cell esds are taken into account individually in the estimation of esds in distances, angles and torsion angles; correlations between esds in cell parameters are only used when they are defined by crystal symmetry. An approximate (isotropic) treatment of cell esds is used for estimating esds involving l.s. planes.
Refinement. Refinement of F^2^ against ALL reflections. The weighted R-factor wR and goodness of fit S are based on F^2^, conventional R-factors R are based on F, with F set to zero for negative F^2^. The threshold expression of F^2^ > 2sigma(F^2^) is used only for calculating R-factors(gt) etc. and is not relevant to the choice of reflections for refinement. R-factors based on F^2^ are statistically about twice as large as those based on F, and R- factors based on ALL data will be even larger.

### Fractional atomic coordinates and isotropic or equivalent isotropic displacement parameters (Å^2^)

**Table:**

	*x*	*y*	*z*	*U*_iso_*/*U*_eq_	Occ. (<1)
S1	0.25448 (11)	0.74526 (3)	0.63308 (5)	0.0389 (2)	
O1	0.1944 (4)	0.86060 (8)	0.40692 (15)	0.0530 (5)	
O2	0.3485 (4)	0.76455 (8)	0.73994 (16)	0.0538 (5)	
O3	0.0155 (3)	0.72397 (9)	0.61106 (17)	0.0559 (6)	
C1	0.2764 (4)	0.80071 (11)	0.5466 (2)	0.0395 (6)	
C2	0.4651 (5)	0.84268 (11)	0.5587 (2)	0.0404 (6)	
C3	0.6729 (5)	0.85332 (12)	0.6334 (2)	0.0448 (7)	
H3	0.7168	0.8296	0.6944	0.054*	
C4	0.8133 (5)	0.89891 (12)	0.6171 (3)	0.0546 (8)	
C5	0.7417 (7)	0.93335 (13)	0.5275 (3)	0.0663 (10)	
H5	0.8380	0.9648	0.5172	0.080*	
C6	0.5384 (7)	0.92401 (13)	0.4532 (3)	0.0668 (10)	
H6	0.4937	0.9479	0.3925	0.080*	
C7	0.4027 (5)	0.87822 (12)	0.4714 (2)	0.0493 (7)	
C8	0.1215 (5)	0.81295 (12)	0.4536 (2)	0.0472 (7)	
C9	1.0410 (5)	0.91196 (14)	0.6956 (3)	0.0679 (10)	
H9A	1.0615	0.8833	0.7537	0.082*	0.546 (8)
H9B	1.1760	0.9081	0.6571	0.082*	0.546 (8)
H9C	1.1325	0.8766	0.7109	0.082*	0.454 (8)
H9D	1.1373	0.9382	0.6602	0.082*	0.454 (8)
C10A	1.0520 (9)	0.96775 (18)	0.7449 (5)	0.0628 (15)	0.546 (8)
H10A	1.2013	0.9697	0.7982	0.075*	0.546 (8)
H10B	1.0655	0.9956	0.6879	0.075*	0.546 (8)
C10B	1.0098 (12)	0.9368 (2)	0.7978 (4)	0.0628 (15)	0.45
H10C	0.9451	0.9071	0.8394	0.075*	0.454 (8)
H10D	1.1694	0.9464	0.8382	0.075*	0.454 (8)
C11	0.8614 (7)	0.98664 (19)	0.7989 (4)	0.1075 (17)	
H11A	0.8464	0.9605	0.8575	0.129*	0.546 (8)
H11B	0.7121	0.9876	0.7470	0.129*	0.546 (8)
H11C	0.8977	1.0245	0.8284	0.129*	0.546 (8)
H11D	0.7007	0.9786	0.7604	0.129*	0.454 (8)
H11E	0.9286	1.0179	0.7633	0.129*	0.454 (8)
H11F	0.8556	0.9971	0.8735	0.129*	0.454 (8)
C12	−0.0968 (5)	0.78663 (15)	0.3948 (3)	0.0608 (9)	
H12A	−0.1150	0.7490	0.4243	0.091*	
H12B	−0.0868	0.7834	0.3183	0.091*	
H12C	−0.2337	0.8099	0.4026	0.091*	
C13	0.4479 (4)	0.69265 (11)	0.5985 (2)	0.0357 (6)	
H13	0.6117	0.7087	0.6103	0.043*	
C14	0.4486 (5)	0.64264 (12)	0.6743 (2)	0.0467 (7)	
H14A	0.4932	0.6554	0.7499	0.056*	
H14B	0.2879	0.6259	0.6648	0.056*	
C15	0.6247 (6)	0.59859 (13)	0.6501 (3)	0.0631 (9)	
H15A	0.7869	0.6145	0.6657	0.076*	
H15B	0.6201	0.5655	0.6975	0.076*	
C16	0.5681 (7)	0.58013 (13)	0.5337 (3)	0.0680 (10)	
H16A	0.4131	0.5605	0.5203	0.082*	
H16B	0.6903	0.5531	0.5193	0.082*	
C17	0.5593 (6)	0.63014 (12)	0.4576 (3)	0.0562 (8)	
H17A	0.5111	0.6170	0.3823	0.067*	
H17B	0.7193	0.6470	0.4648	0.067*	
C18	0.3844 (5)	0.67500 (12)	0.4819 (2)	0.0460 (7)	
H18A	0.3900	0.7081	0.4347	0.055*	
H18B	0.2212	0.6596	0.4672	0.055*	

### Atomic displacement parameters (Å^2^)

**Table:**

	*U*^11^	*U*^22^	*U*^33^	*U*^12^	*U*^13^	*U*^23^
S1	0.0385 (4)	0.0493 (4)	0.0324 (4)	−0.0019 (3)	0.0152 (3)	−0.0023 (3)
O1	0.0688 (13)	0.0535 (13)	0.0380 (12)	0.0149 (10)	0.0126 (10)	0.0054 (9)
O2	0.0718 (14)	0.0603 (13)	0.0316 (12)	−0.0018 (10)	0.0157 (9)	−0.0065 (9)
O3	0.0342 (10)	0.0742 (15)	0.0638 (15)	−0.0046 (9)	0.0214 (9)	0.0022 (11)
C1	0.0403 (14)	0.0445 (16)	0.0359 (16)	0.0066 (12)	0.0130 (11)	−0.0059 (12)
C2	0.0498 (15)	0.0363 (15)	0.0394 (16)	0.0060 (12)	0.0192 (13)	−0.0045 (12)
C3	0.0507 (16)	0.0402 (16)	0.0469 (18)	0.0030 (13)	0.0179 (13)	−0.0075 (13)
C4	0.0608 (18)	0.0431 (18)	0.066 (2)	−0.0014 (14)	0.0290 (16)	−0.0147 (15)
C5	0.086 (2)	0.045 (2)	0.077 (3)	−0.0140 (17)	0.038 (2)	−0.0097 (18)
C6	0.106 (3)	0.0427 (19)	0.059 (2)	0.0026 (18)	0.034 (2)	0.0077 (16)
C7	0.0641 (19)	0.0447 (17)	0.0431 (18)	0.0082 (14)	0.0202 (15)	−0.0015 (14)
C8	0.0496 (16)	0.0544 (18)	0.0411 (18)	0.0119 (14)	0.0173 (13)	−0.0030 (14)
C9	0.0515 (18)	0.061 (2)	0.096 (3)	−0.0124 (16)	0.0261 (18)	−0.0307 (19)
C10A	0.068 (3)	0.044 (3)	0.077 (4)	−0.010 (2)	0.013 (2)	−0.011 (2)
C10B	0.068 (3)	0.044 (3)	0.077 (4)	−0.010 (2)	0.013 (2)	−0.011 (2)
C11	0.093 (3)	0.119 (4)	0.120 (4)	−0.016 (3)	0.043 (3)	−0.060 (3)
C12	0.0468 (16)	0.090 (2)	0.0434 (19)	0.0127 (17)	0.0029 (13)	−0.0030 (17)
C13	0.0329 (12)	0.0409 (15)	0.0348 (15)	−0.0021 (11)	0.0105 (11)	0.0010 (11)
C14	0.0567 (17)	0.0467 (17)	0.0378 (17)	−0.0057 (13)	0.0114 (13)	0.0059 (13)
C15	0.077 (2)	0.0461 (19)	0.068 (3)	0.0101 (16)	0.0184 (18)	0.0145 (16)
C16	0.092 (2)	0.0427 (18)	0.075 (3)	0.0051 (17)	0.029 (2)	−0.0013 (17)
C17	0.074 (2)	0.0478 (18)	0.052 (2)	0.0056 (15)	0.0261 (16)	−0.0057 (15)
C18	0.0571 (17)	0.0480 (17)	0.0353 (17)	0.0038 (13)	0.0147 (13)	0.0005 (13)

### Geometric parameters (Å, °)

**Table:**

S1—O2	1.434 (2)	C10B—H10C	0.9900
S1—O3	1.4401 (19)	C10B—H10D	0.9900
S1—C1	1.728 (3)	C11—H11A	0.9800
S1—C13	1.775 (3)	C11—H11B	0.9800
O1—C8	1.374 (3)	C11—H11C	0.9800
O1—C7	1.383 (4)	C11—H11D	0.9800
C1—C8	1.369 (4)	C11—H11E	0.9800
C1—C2	1.458 (4)	C11—H11F	0.9800
C2—C7	1.382 (4)	C12—H12A	0.9800
C2—C3	1.402 (4)	C12—H12B	0.9800
C3—C4	1.386 (4)	C12—H12C	0.9800
C3—H3	0.9500	C13—C18	1.510 (4)
C4—C5	1.395 (5)	C13—C14	1.523 (4)
C4—C9	1.522 (4)	C13—H13	1.0000
C5—C6	1.376 (5)	C14—C15	1.521 (4)
C5—H5	0.9500	C14—H14A	0.9900
C6—C7	1.379 (4)	C14—H14B	0.9900
C6—H6	0.9500	C15—C16	1.511 (5)
C8—C12	1.474 (4)	C15—H15A	0.9900
C9—C10A	1.458 (3)	C15—H15B	0.9900
C9—C10B	1.458 (3)	C16—C17	1.521 (4)
C9—H9A	0.9900	C16—H16A	0.9900
C9—H9B	0.9900	C16—H16B	0.9900
C9—H9C	0.9900	C17—C18	1.530 (4)
C9—H9D	0.9900	C17—H17A	0.9900
C10A—C11	1.458 (3)	C17—H17B	0.9900
C10A—H10A	0.9900	C18—H18A	0.9900
C10A—H10B	0.9900	C18—H18B	0.9900
C10B—C11	1.457 (3)		
			
O2—S1—O3	118.49 (12)	C10B—C11—H11A	68.0
O2—S1—C1	107.06 (13)	C10A—C11—H11A	109.5
O3—S1—C1	108.66 (13)	C10B—C11—H11B	117.0
O2—S1—C13	108.04 (12)	C10A—C11—H11B	109.5
O3—S1—C13	108.74 (12)	H11A—C11—H11B	109.5
C1—S1—C13	105.04 (12)	C10B—C11—H11C	131.4
C8—O1—C7	107.1 (2)	C10A—C11—H11C	109.5
C8—C1—C2	107.5 (2)	H11A—C11—H11C	109.5
C8—C1—S1	126.4 (2)	H11B—C11—H11C	109.5
C2—C1—S1	126.2 (2)	C10B—C11—H11D	109.6
C7—C2—C3	119.5 (3)	C10A—C11—H11D	115.2
C7—C2—C1	104.6 (3)	H11A—C11—H11D	92.7
C3—C2—C1	135.9 (3)	H11C—C11—H11D	118.9
C4—C3—C2	119.1 (3)	C10B—C11—H11E	109.3
C4—C3—H3	120.4	C10A—C11—H11E	68.2
C2—C3—H3	120.4	H11A—C11—H11E	156.6
C3—C4—C5	118.9 (3)	H11B—C11—H11E	92.6
C3—C4—C9	121.0 (3)	H11C—C11—H11E	53.7
C5—C4—C9	120.1 (3)	H11D—C11—H11E	109.5
C6—C5—C4	123.2 (3)	C10B—C11—H11F	109.5
C6—C5—H5	118.4	C10A—C11—H11F	133.2
C4—C5—H5	118.4	H11A—C11—H11F	54.0
C5—C6—C7	116.6 (3)	H11B—C11—H11F	117.3
C5—C6—H6	121.7	H11C—C11—H11F	56.3
C7—C6—H6	121.7	H11D—C11—H11F	109.5
C6—C7—C2	122.7 (3)	H11E—C11—H11F	109.5
C6—C7—O1	126.4 (3)	C8—C12—H12A	109.5
C2—C7—O1	110.9 (3)	C8—C12—H12B	109.5
C1—C8—O1	109.9 (3)	H12A—C12—H12B	109.5
C1—C8—C12	134.6 (3)	C8—C12—H12C	109.5
O1—C8—C12	115.4 (3)	H12A—C12—H12C	109.5
C10A—C9—C4	115.4 (4)	H12B—C12—H12C	109.5
C10B—C9—C4	115.5 (3)	C18—C13—C14	111.7 (2)
C10A—C9—H9A	108.4	C18—C13—S1	112.51 (18)
C10B—C9—H9A	68.9	C14—C13—S1	108.88 (17)
C4—C9—H9A	108.4	C18—C13—H13	107.9
C10A—C9—H9B	108.4	C14—C13—H13	107.9
C10B—C9—H9B	134.7	S1—C13—H13	107.9
C4—C9—H9B	108.4	C15—C14—C13	109.6 (2)
H9A—C9—H9B	107.5	C15—C14—H14A	109.8
C10A—C9—H9C	134.9	C13—C14—H14A	109.8
C10B—C9—H9C	108.4	C15—C14—H14B	109.8
C4—C9—H9C	108.5	C13—C14—H14B	109.8
H9B—C9—H9C	65.3	H14A—C14—H14B	108.2
C10A—C9—H9D	68.8	C16—C15—C14	111.1 (3)
C10B—C9—H9D	108.2	C16—C15—H15A	109.4
C4—C9—H9D	108.5	C14—C15—H15A	109.4
H9A—C9—H9D	139.7	C16—C15—H15B	109.4
H9C—C9—H9D	107.5	C14—C15—H15B	109.4
C11—C10A—C9	120.0 (3)	H15A—C15—H15B	108.0
C11—C10A—H9D	151.9	C15—C16—C17	111.4 (3)
C11—C10A—H10A	107.3	C15—C16—H16A	109.3
C9—C10A—H10A	107.3	C17—C16—H16A	109.3
H9D—C10A—H10A	99.2	C15—C16—H16B	109.3
C11—C10A—H10B	107.3	C17—C16—H16B	109.3
C9—C10A—H10B	107.3	H16A—C16—H16B	108.0
H9D—C10A—H10B	72.7	C16—C17—C18	111.3 (2)
H10A—C10A—H10B	106.9	C16—C17—H17A	109.4
C9—C10A—H11E	147.4	C18—C17—H17A	109.4
H9D—C10A—H11E	140.8	C16—C17—H17B	109.4
H10A—C10A—H11E	104.2	C18—C17—H17B	109.4
H10B—C10A—H11E	70.6	H17A—C17—H17B	108.0
C11—C10B—C9	120.0 (3)	C13—C18—C17	109.6 (2)
C11—C10B—H10C	107.3	C13—C18—H18A	109.7
C9—C10B—H10C	107.3	C17—C18—H18A	109.7
C11—C10B—H10D	107.3	C13—C18—H18B	109.7
C9—C10B—H10D	107.3	C17—C18—H18B	109.7
H10C—C10B—H10D	106.9	H18A—C18—H18B	108.2
			
O2—S1—C1—C8	−146.3 (2)	S1—C1—C8—C12	−1.6 (5)
O3—S1—C1—C8	−17.2 (3)	C7—O1—C8—C1	1.0 (3)
C13—S1—C1—C8	99.0 (2)	C7—O1—C8—C12	−178.5 (2)
O2—S1—C1—C2	34.5 (2)	C3—C4—C9—C10A	−121.6 (4)
O3—S1—C1—C2	163.6 (2)	C5—C4—C9—C10A	57.8 (4)
C13—S1—C1—C2	−80.2 (2)	C3—C4—C9—C10B	−74.6 (4)
C8—C1—C2—C7	1.6 (3)	C5—C4—C9—C10B	104.8 (4)
S1—C1—C2—C7	−179.10 (19)	C10B—C9—C10A—C11	−48.1 (4)
C8—C1—C2—C3	−179.1 (3)	C4—C9—C10A—C11	52.7 (7)
S1—C1—C2—C3	0.2 (4)	C10A—C9—C10B—C11	48.2 (4)
C7—C2—C3—C4	−1.0 (4)	C4—C9—C10B—C11	−52.2 (7)
C1—C2—C3—C4	179.8 (3)	C9—C10B—C11—C10A	−48.1 (4)
C2—C3—C4—C5	0.9 (4)	C9—C10A—C11—C10B	48.1 (4)
C2—C3—C4—C9	−179.7 (2)	O2—S1—C13—C18	−172.09 (18)
C3—C4—C5—C6	−0.7 (5)	O3—S1—C13—C18	58.1 (2)
C9—C4—C5—C6	179.9 (3)	C1—S1—C13—C18	−58.1 (2)
C4—C5—C6—C7	0.5 (5)	O2—S1—C13—C14	63.6 (2)
C5—C6—C7—C2	−0.5 (4)	O3—S1—C13—C14	−66.2 (2)
C5—C6—C7—O1	−179.1 (3)	C1—S1—C13—C14	177.59 (18)
C3—C2—C7—C6	0.8 (4)	C18—C13—C14—C15	58.5 (3)
C1—C2—C7—C6	−179.7 (3)	S1—C13—C14—C15	−176.6 (2)
C3—C2—C7—O1	179.5 (2)	C13—C14—C15—C16	−56.8 (3)
C1—C2—C7—O1	−1.0 (3)	C14—C15—C16—C17	55.9 (4)
C8—O1—C7—C6	178.8 (3)	C15—C16—C17—C18	−55.2 (4)
C8—O1—C7—C2	0.1 (3)	C14—C13—C18—C17	−57.8 (3)
C2—C1—C8—O1	−1.6 (3)	S1—C13—C18—C17	179.46 (18)
S1—C1—C8—O1	179.08 (17)	C16—C17—C18—C13	55.5 (3)
C2—C1—C8—C12	177.8 (3)		

### Hydrogen-bond geometry (Å, °)

**Table:**

Cg is the centroid of the C2–C7 benzene ring.

**Table:**

*D*—H···*A*	*D*—H	H···*A*	*D*···*A*	*D*—H···*A*
C13—H13···O3^i^	1.00	2.34	3.314 (3)	164
C18—H18A···O2^ii^	0.99	2.51	3.345 (4)	142
C12—H12C···Cg^iii^	0.98	2.84	3.659 (3)	141

Symmetry codes: (i) *x*+1, *y*, *z*; (ii) *x*, −*y*+3/2, *z*−1/2; (iii) *x*−1, *y*, *z*.
